# Vitamin D Deficiency Is Associated with Advanced Liver Fibrosis and Impaired Fasting Glucose in Alcohol Use Disorder

**DOI:** 10.3390/nu16081099

**Published:** 2024-04-09

**Authors:** Paola Zuluaga, Julia Casado-Carbajo, Anna Hernández-Rubio, Marvin Bueno-Vélez, Carmen García-Martin, Robert Muga, Daniel Fuster

**Affiliations:** 1 Addiction Unit, Department of Internal Medicine, Hospital Universitari Germans Trias i Pujol, 08916 Badalona, Spain; ypzuluaga.germanstrias@gencat.cat (P.Z.); julia.casado.carbajo@gmail.com (J.C.-C.); anna.hernandez.rubio@gmail.com (A.H.-R.); marvinbueno96@gmail.com (M.B.-V.); rmuga.germanstrias@gencat.cat (R.M.); 2 Department of Medicine, Universitat Autònoma de Barcelona, 08916 Badalona, Spain; 3 Spanish Society of Internal Medicine-SEMI-“Alcohol and Other Drugs” Work Group, 28016 Madrid, Spain; 4 Laboratori Clinic Metropolitana Nord, Department of Biochemistry, Hospital Universitari Germans Trias i Pujol, 08916 Badalona, Spain; cgarciama.germanstrias@gencat.cat

**Keywords:** vitamin D, alcohol use disorder, fasting glucose, advanced liver fibrosis

## Abstract

Background: Vitamin D deficiency is a risk factor for liver disease, insulin resistance, and beta cell dysfunction. Individuals with alcohol use disorder (AUD) have many comorbidities, with a heavy burden of liver disease and metabolic complications, including type 2 diabetes mellitus (T2DM). Objective: We aimed to analyze the prevalence and associations of vitamin D deficiency in patients admitted for in-hospital treatment of AUD. Methods: A cross-sectional study was conducted in patients consecutively admitted for the treatment of AUD between January 2017 and October 2023. Sociodemographic data, substance use characteristics, and blood parameters were available at admission. Vitamin D status was assessed through the serum concentrations of 25-hydroxyvitamin D [25(OH)D] levels using a direct competitive chemiluminescent immunoassay method. Deficiency of vitamin D was defined as a concentration less than 20 ng/mL; impaired fasting glucose (IFG) was defined by fasting blood glucose >100 mg/dL (5.6 mmol/L), and advanced liver fibrosis by an FIB-4 index >3.25. Results: Two hundred and forty-three patients were included (75% male) with a mean age of 49 ± 10 years, mean BMI of 26.4 ± 7.3, mean alcohol consumption of 163 ± 81 g/day, and a mean duration of AUD of 18.1 ± 11.2 years. Mean 25(OH)D, fasting blood glucose, AST, ALT, and platelets were 14.4 ± 10.2 ng/mL, 103.4 ± 40.9 mg/dL, 55.1 ± 75.8 U/L, 44.8 ± 76.6 U/L, and 206.3 ± 84.8 × 10^9^/L, respectively. The prevalence of vitamin D deficiency was 80.6%, and 41.1% of patients had levels less than 10 ng/mL. IFG was present in 32.3% of patients, and 20.5% had FIB-4 values >3.25. In the multivariable analysis, IFG (OR, 2.51; 95% CI: 1.02–6.17, *p* = 0.04) and advanced liver fibrosis (OR, 4.27; 95% CI: 1.21–15.0, *p* = 0.02) were the only factors associated with vitamin D deficiency. Conclusions: Vitamin D deficiency was very prevalent in this series of patients with AUD and was associated with impaired fasting glucose and advanced liver fibrosis.

## 1. Introduction

Vitamin D plays an essential role in maintaining bone health, and it also has immunomodulatory properties and is an independent predictor of risk for cancer and other chronic diseases such as metabolic syndrome [1,2]. The serum concentration of 25-hydroxyvitamin D [25(OH)D], but not that of 1.25 dihydroxyvitamin D, defines nutritional vitamin D status. Vitamin D is involved in the pathogenesis of type 2 diabetes mellitus (T2DM), with a positive correlation of vitamin D concentrations with insulin sensitivity index and beta cell function [3]. In addition to the higher prevalence in people with T2DM, vitamin D deficiency is associated with cardiovascular events and mortality among adults with prediabetes and diabetes [4].

Vitamin D contributes to liver physiology, a factor implicated in the regulation of homeostasis, inflammation, and liver fibrogenesis [5]. The association between chronic liver disease and vitamin D deficiency is well established, especially in patients with end-stage liver disease [6,7]. Low serum 25(OH)D levels are associated with advanced liver disease in the general population [8] and with poor prognosis and mortality in patients with advanced liver disease [9,10,11].

Alcohol use disorder (AUD) is one of the most prevalent substance use disorders worldwide [12]. In addition to the heavy burden of liver disease and metabolic syndrome in people with AUD [13], chronic exposure to alcohol has an impact on bone metabolism with an increased risk of osteoporosis and vertebral fractures, as demonstrated in clinical and in vitro studies [14]. However, studies of the prevalence and clinical associations of vitamin D deficiency in people who consume alcohol have shown mixed results [15]. A review by Tardelli and colleagues described the association between the level of vitamin D and alcohol intake [15]. Some of the studies included in the review found a positive association but others found a negative or no association between alcohol use levels and vitamin D levels [15]. In addition, the studies differed in their purposes and in the methods they used to detect vitamin D metabolites and to quantify alcohol consumption [15].

Despite the association of vitamin D deficiency with the progression of liver disease or with metabolic syndrome, the impact of vitamin D deficiency in the comorbidity of patients with AUD with no clinically apparent alcohol-associated liver disease is not well established. In addition, there is a lack of high-quality studies regarding nutrition in patients with unhealthy alcohol use. Therefore, the main objective of this study was to analyze the prevalence and clinical associations of vitamin D deficiency in patients seeking hospital treatment for AUD without evidence of end-stage liver disease.

## 2. Materials and Methods

### 2.1. Design and Study Population

This was a cross-sectional study in a series of patients with severe AUD admitted for inpatient detoxification at Hospital Universitari Germans Trias I Pujol in Badalona, Spain, in the period January 2017 through October 2023. Patients included had severe AUD according to the Diagnostic and Statistical Manual of Mental Disorders (DSM)-5 criteria. Addiction specialists at the primary care level referred patients to hospital detoxification. The inclusion criteria for referring patients to hospital detoxification were as follows: high risk for severe alcohol withdrawal (i.e., previous episodes of severe alcohol withdrawal and/or prior history of seizures or delirium tremens or multiple prior attempts of alcohol detoxification); the presence of other chronic medical comorbidities; and history of multiple unsuccessful outpatient alcohol detoxifications. Exclusion criteria included severe psychiatric comorbidities and the presence of acute medical comorbidities (i.e., acute infection or decompensated liver disease). All patients provided written informed consent before entering the study. The methods of this study complied with ethical standards for medical research and the principles of good clinical practice in accordance with the World Medical Association’s Declaration of Helsinki. In addition, we adhered to the STROBE checklist for cross-sectional studies (Supplementary Materials).

### 2.2. Measurements

On the first day of admission, we performed a comprehensive interview to register sociodemographic data and history of alcohol use, including type and amount of alcohol consumed, duration of the disorder, age when alcohol consumption began, and use of other legal and illegal drugs. We also performed a thorough physical examination including measurement of weight and height to calculate body mass index (BMI). We further stratified BMI as below 25 kg/m^2^ or greater than 25 kg/m^2^, which is consistent with being overweight. On the second day of admission, we drew blood samples to assess general hematological and biochemical parameters that included liver and bone tests.

Vitamin D status was assessed through the serum level of 25(OH)D, measured by a Liaison^®^ 25 OH assay (Diasorin, Saluggia, Italy), by the automated direct competitive chemiluminescence immunoassay (CLIA) method. In the first incubation, 25(OH)D dissociated from its binding protein and bound to the specific antibody in the solid phase. After 10 min the tracer, [25(OH)D linked to an isoluminol derivative] was added. After a second 10 min incubation, the unbound material was removed with a wash cycle. Subsequently, the starter reagents were added to initiate a flash chemiluminescent reaction. The light signal was measured by a photomultiplier as relative light units (RLUs) and was inversely proportional to the concentration of 25(OH)D present in the sample. The method has an Inter-Assay CV <10% (8.8) and the performance and quality measurements of the results are guaranteed by UKNEQAS and FPCQLC external control quality programs. We compared these results with other methods by external quality control.

According to the International Osteoporosis Foundation, we defined the levels of 25(OH)D as follows: undetectable (concentration less than 4 ng/mL), severe deficit (concentrations less than 10 ng/mL), deficit (concentrations less than 20 ng/mL), and adequate levels (concentrations more than 20 ng/mL). Associations with different clinical parameters were made with concentrations of 25(OH)D less than 20 ng/mL, which are widely accepted as a threshold for deficiency that is associated with unfavorable health outcomes [16].

We estimated liver fibrosis using the FIB-4 index, which was first described by Sterling and colleagues [17] and is calculated as follows: age × AST (U/L)/platelet count (10^9^/L) × ALT (U/L)^1/2^.

Advanced liver fibrosis was defined as a FIB-4 index greater than 3.25, and impaired fasting glucose was defined as fasting blood glucose >100 mg/dL (5.6 mmol/L). In addition, we defined the different laboratory abnormalities as values that were out of the reference range of the center’s laboratory.

### 2.3. Statistical Analysis

We expressed descriptive statistics as the median (interquartile range [IQR]) and mean ± standard deviation for quantitative variables and as absolute frequencies and percentages for qualitative variables. We performed bivariate analyses to establish differences between the different levels of 25(OH)D using the chi-square, F-Fisher, Mann–Whitney U, ANOVA, and Kruskal–Wallis tests where appropriate. We used logistic regression models to find associations with vitamin D deficiency. Variables with statistically significant associations with vitamin D deficiency in the univariate analysis were included in the multivariable analysis in a single step. We also adjusted the multivariable model by age and alcohol consumption. We considered *p*-values *<* 0.05 statistically significant. We performed statistical analysis using Stata software (version 11.0; College Station, TX, USA).

## 3. Results

We included 243 patients (75% males) with a mean age of 49 ± 10 years and a mean body mass index (BMI) of 26.4 ± 7.3. The mean alcohol consumption was 163 ± 81 g/day and the mean duration of AUD was 18.1 ± 11.2 years. The mean levels of fasting blood glucose, AST, ALT, and platelets were 103.4 ± 40.9 mg/dL, 55.1 ± 75.8 U/L, 44.8 ± 76.6 U/L, and 206.3 ± 84.8 × 10^9^/L, respectively.

Table 1 shows the clinical and sociodemographic characteristics as well as the main laboratory parameters of the study participants.

### 3.1. 25(OH)D Levels and Bivariate Associations of Vitamin D Deficiency

Mean 25(OH)D levels were 14.4 ± 10.2 ng/mL, with a similar distribution between men and women (14.3 ± 9.5 ng/mL vs. 14.5 ± 12.3 ng/mL, respectively) and among patients with or without overweight/obesity according to BMI (13.8 ± 8.7 ng/mL vs. 15.1 ± 12.4 ng/mL, respectively). A vast majority of patients (80.6%) presented 25(OH)D levels <20 ng/mL, while 41.1% presented 25(OH)D levels <10 ng/mL, and 4% presented 25(OH)D levels below the limit of detection (4 ng/mL).

In patients with FIB-4 suggestive of advanced liver fibrosis, the prevalence of vitamin D deficit was 94% and of severe deficit was 52%. The mean value of 25(OH)D levels was lower in patients with FIB-4 suggestive of advanced liver fibrosis (10.8 ± 5.2 ng/mL vs. 15.3 ± 11 ng/mL, respectively; *p* < 0.01) and was also lower among patients with impaired fasting glucose levels (12.2 ± 6.6 ng/mL vs. 15.4 ± 11.5 ng/mL, respectively; *p* = 0.02).

Table 2 shows the main clinical and sociodemographic characteristics and laboratory parameters stratified by 25(OH)D levels. Table 2 includes the dispersion of continuous variables as well as the prevalence of abnormal values.

### 3.2. 25(OH)D Levels and Bone Metabolism Markers

Among all patients, 25% presented hypomagnesemia, 18.9% presented hypocalcemia, 2.1% presented hypophosphatemia, and 27.6% had high parathyroid hormone (PTH) levels. The median PTH values were significantly higher among patients with vitamin D deficiency (62.8 ± 31.9 pg/mL vs. 47 ± 22.8 pg/mL, respectively; *p* < 0.01).

Table 3 shows the main laboratory results stratified by 25(OH) levels.

### 3.3. Associations of Clinical and Laboratory Parameters with Vitamin D Deficiency

In the univariate model, vitamin D deficiency was associated with age greater than 50 (*p* = 0.02), impaired fasting glucose (*p* = 0.02), and the presence of advanced liver fibrosis (*p* = 0.01). In the multivariable analysis, adjusted by age and alcohol consumption, impaired fasting glucose (OR 2.51, CI 95%: 1.02–6.17, *p* = 0.04) and advanced liver fibrosis (OR 4.27, CI 1.21–15.0, *p* = 0.02) were the only factors associated with vitamin D deficiency. Table 4 shows the univariate and multivariable regression analyses.

## 4. Discussion

In this study, 80% of patients with AUD admitted for hospital treatment of the disorder presented vitamin D deficiency, which was severe in 40% of the cases. The presence of FIB-4 values suggestive of advanced liver fibrosis and/or impaired fasting glucose was independently associated with vitamin D deficiency in this group of patients. In addition, one-third of the patients included in this study who had vitamin D deficiency also had high PTH levels.

There are several mechanisms that have been proposed as potential explanations for low vitamin D levels in patients with AUD, including poor diet, impaired liver hydroxylation, decreased intestinal absorption due to cholestasis or pancreatic insufficiency, reduced hepatic production of proteins that help to fix vitamin D, chronic inflammation, and reduced solar exposure [15].

In this study, the prevalence of vitamin D deficiency was greater than the prevalence found in other series of treatment-seeking patients with AUD, as studies from Asia and Northern and Central Europe reported a prevalence ranging from 60% and 75% [18,19,20,21,22]. A recent Spanish study among patients with severe AUD admitted to an internal medicine ward in the Canary Islands reported a prevalence of vitamin D deficiency of 28% [23].

The prevalence of vitamin D deficiency among patients with FIB-4 values suggestive of advanced liver fibrosis in this study was almost universal (94% of patients with FIB-4 values >3.25 presented vitamin levels <20 ng/mL and 52% presented levels <10 ng/mL). These results are similar to those reported by Anty and colleagues, as 86% of their patients with chronic liver disease presented 25(OH)D levels <20 ng/mL and 60.4% had levels <10 ng/mL [24].

We also report the association between FIB-4 values suggestive of advanced liver fibrosis, impaired fasting glucose, and vitamin D deficiency. Vitamin D has pleiotropic actions in immune modulation and affects chronic inflammation, insulin resistance, and chronic liver disease [1]. Vitamin D also reduces the secretion of pro-inflammatory cytokines, increases anti-inflammatory cytokine production, and reduces lymphocyte migration and activation in adipose tissue, contributing to a reduction in insulin resistance [25]. In addition, vitamin D decreases the synthesis of inflammatory proteins such as matrix metalloproteinase (MMP) and *C*-reactive protein (CRP) in chronic inflammation [26]. Also, the activation of vitamin D receptors in liver macrophages decreases liver inflammation, steatosis, and insulin resistance [27].

Despite the implication of vitamin D deficiency in several pathological processes, the screening and treatment in the general population and in people with alcohol use disorder without evidence of advanced liver disease are still a matter of debate. Initial results from meta-analyses of randomized controlled trials with vitamin D supplementation have demonstrated lower mortality in the vitamin D-treated group than in the placebo group [28,29]. A recent real-world study has shown that routine determination of vitamin D and its supplementation in people with low levels are practices that impact global mortality [30]. However, other studies have shown no benefit from vitamin D supplementation in several poor health outcomes, including the risk of fractures, the occurrence of invasive cancer, or incident cardiovascular events [31,32]. The results of randomized clinical trials examining the effect of vitamin D supplementation on liver disease have been inconclusive. The last review of vitamin D supplementation in chronic liver disease by the Cochrane Hepato-Biliary Group concluded that the certainty of the evidence for all outcomes is very low [33]. Despite the lack of evidence, the 2019 guidelines of the European Association for the Study of the Liver (EASL) recommend the measurement of 25(OH)D levels and supplementation, if needed, in patients with chronic liver disease, especially in those with cholestasis, cirrhosis, steatosis, or levels below 20 ng/mL [34]. Of note, following EASL recommendations, all patients included in this study group received vitamin D supplementation in the form of calcifediol if 25(OH)D levels were below 20 ng/mL. Low 25(OH)D levels in patients with unhealthy alcohol use might reflect a true nutritional deficit, which is usually associated with other vitamin (thiamine, pyridoxine, folate, and cobalamin) deficits [34].

Another notable finding of our study is the prevalence of high PTH levels in patients with vitamin D deficiency. High PTH is associated with increased bone resorption, which acts to regain serum calcium levels. Over time, the presence of high PTH levels can lead to reduced bone mineral density, as bone formation is unable to equal the rates of resorption, increasing the risk of osteoporosis and fractures [35]. This condition could increase the susceptibility of bone density loss and fractures in these already vulnerable patients [14]. These findings suggest the importance of maintaining adequate levels of vitamin D to avoid stimulating the parathyroid gland in a population that is already at high risk for accidents and injuries.

Of note, the median GGT value in the study group was 105 UI/L. In a prior study by our group, 75% of the 728 patients admitted for hospital detoxification had GGT values >50 UI/L [36]. GGT is not only a marker of excessive alcohol use but also of intracellular triglyceride levels in the liver, and it is associated with being overweight, insulin resistance, and atherosclerosis. Therefore, the patients included in this study are at risk of several poor clinical outcomes.

We have to note several limitations of this study. First, it is cross-sectional in nature, so we are not able to assign causality to the associations found. Second, we estimated advanced liver fibrosis with the use of FIB-4 because transient elastography was not available for the majority of patients. Despite not being ideal, FIB-4 has been used to estimate liver fibrosis in patients in whom the performance of a liver biopsy is unlikely, and in settings where other, more expensive tests are not available [37]. Third, we included patients at the higher end of the spectrum of AUD severity, and our results may not be generalizable to patients seen in primary care with milder forms of unhealthy alcohol use. Fourth, in this study, we used the chemiluminescent immunoassay method to assess 25(OH)D levels instead of mass spectrometry.

On the other hand, this study also has important strengths: the number of patients included is high, the prevalence of vitamin D deficiency is also very high, and the associations found are relevant from a clinical standpoint and consistent with other studies in the literature.

Our results underscore the high prevalence of vitamin D deficiency in patients with AUD, a deficiency that is almost universal in those with advanced liver fibrosis. The benefits of measuring 25(OH)D levels and treating vitamin D deficiency go beyond bone health and may have an impact on the prognosis of both liver disease and insulin resistance [38]. Our results also stress the importance of other co-occurring disorders, like overweight/obesity and insulin resistance, in a population that is already at high risk of advanced liver disease [13]. Future studies around vitamin D values in patients with unhealthy alcohol use could include the analysis of polymorphisms of the vitamin D receptors (VDRs) to investigate their impact on the pathogenesis and progression of liver steatosis [27].

In addition, our findings speak to the need for an accurate nutritional assessment in this patient setting, that is, individuals with unhealthy alcohol use at risk for the development of progressive liver disease [34].

Therefore, promoting abstinence from alcohol consumption can have benefits that go beyond the mitigation of alcohol-associated liver disease progression. In fact, abstinence could impact bone density loss [39] and the occurrence of metabolic syndrome [40].

## 5. Conclusions

Vitamin D deficiency was very prevalent in this series of patients with AUD and was associated with the presence of impaired fasting glucose and/or the presence of FIB-4 values suggestive of advanced liver fibrosis.

## Figures and Tables

**Table 1:** Sociodemographic and basal laboratory characteristics in 243 AUD patients.

		Reference Values
Age at baseline, median [IQR], years	50 [41–57]	-
Male, (%)	184 (75.7)	
BMI, median [IQR], kg/m^2^, *n* = 213	25.1 [22.1–29.3]	
Alcohol consumption, median [IQR], g/day	150 [100–200]	
Duration of AUD, median [IQR], years	17 [10–25]	
Fasting blood glucose, median [IQR], mg/dL, *n* = 241	94 [86–104]	70–100
Triglycerides, median [IQR], mg/dL, *n* = 241	117 [77–180]	<150
Cholesterol, median [IQR], mg/dL, *n* = 241	191 [159–225]	<200
Calcium, median [IQR], mg/dL, *n* = 238	9.2 [8.9–9.5]	8.8–10.6
Phosphate, median [IQR], mg/dL, *n* = 238	3.8 [3.4–4.2]	2.5–4.5
Magnesium, median [IQR], mg/dL, *n* = 236	1.9 [1.8–2]	1.8–2.6
Creatinine, median [IQR], mg/dL, *n* = 242	0.75 [0.65–0.87]	0.72–1.18
GGT, median [IQR], U/L, *n* = 241	105 [34–271]	0–50
Alkaline phosphatase, median [IQR], U/L, *n* = 242	86 [67–105]	30–120
Parathyroid hormone, median [IQR], pg/mL, *n* = 206	52.1 [40.7–72.1]	15–68.3
Albumin, median [IQR], g/L, *n* = 241	37.8 [35–39.8]	35–52
FIB-4 index, median [IQR], *n* = 241	1.4 [0.91–2.8]	<1.45

GGT, gamma glutamyl transpeptidase.

**Table 2:** Sociodemographic characteristics and prevalence of alterations in 243 patients with AUD according to 25(OH)D levels.

	25(OH)D Levels <10 ng/mL (*n* = 100)	25(OH)D Levels 10–20 ng/mL (*n* = 96)	25(OH)D Levels >20 ng/mL (*n* = 47)	*p*
Age at baseline, median [IQR], years Age > 50	52 [43–57.5] 61 (61)	49 [40–56] 47 (48.9)	45 [41–53] 17 (36.1)	0.02 0.01
Male, (%)	76 (76)	74 (77)	34 (72)	0.82
BMI >25, (%)	25.6 [21.6–29.2] 49 (54.4)	25.3 [22.7–29.7] 46 (56.1)	24.9 [22.6–28.3] 20 (48.7)	0.55 0.74
Alcohol consumption, median [IQR], g/day	140 [100–200]	150 [100–200]	160 [120–200]	0.81
Glucose, mg/dL Impaired fasting glucose, (%)	93 [86–106] 35 (35.3)	94.5 [87–107] 35 (36.4)	92.5 [86–98] 8 (17.3)	0.19 0.05
Albumin, mg/dL	37.3 [34.7–39.7]	37.7 [35.7–39.7]	38.6 [35.8–40.8]	0.21
Triglycerides, mg/dL >150 (%)	112 [72.5–180] 34 (34)	135 [78–181.5] 38 (39.5)	128 [105–177] 17 (37.7)	0.98 0.71
Cholesterol >200 (%)	196 [162–237] 42 (42)	186 [161–217] 36 (37.5)	185.5 [146–213] 18 (39.1)	0.42 0.77
FIB-4 >3.25 (%)	1.8 [1–3.3] 26 (26.2)	1.4 [0.9–2.7] 21 (21.8)	1 [0.7–1.8] 3 (6.5)	0.02 0.02
GGT, U/L GGT > 50 (%)	136 [47.5–336.5] 74 (74)	102 [32–274] 64 (67.3)	57 [31–133] 28 (60)	0.01 0.25
Creatinine, mg/dL	0.71 [0.61–0.8]	0.78 [0.67–0.91]	0.82 [0.71–0.92]	0.02

BMI, body mass index; GGT, gamma glutamyl transpeptidase. Values are reported as numbers and percentages or as means with standard deviation.

**Table 3:** Bone metabolism characteristics and prevalence of alterations in 243 patients with AUD according to 25(OH)D levels.

	25(OH)D Levels <10 ng/mL (*n* = 100)	25(OH)D Levels 10–20 ng/mL (*n* = 96)	25(OH)D Levels > 20 ng/mL (*n* = 47)	*p*
Magnesium, mg/dL <1.8 (%)	1.9 [1.8–2.1] 24 (24.7)	1.8 [1.7–2] 27 (29)	1.9 [1.8–2] 8 (17.3)	0.37 0.32
Phosphate, mg/dL <2.5 (%)	3.8 [3.4–4.2] 1 (1)	3.8 [3.5–4.2] 2 (2.1)	3.8 [3.4–4.3] 2 (4.4)	0.44 0.41
Calcium, mg/dL <8.8 (%)	9.2 [8.9–9.4] 20 (20.6)	9.2 [8.9–9.5] 16 (17)	9.3 [9–9.8] 9 (19.1)	0.15 0.81
Alkaline phosphatase, U/L >120 (%)	82 [62.5–112.5] 22 (22)	88 [72–103.5] 16 (16.6)	83.5 [67–99] 4 (8.7)	0.57 0.13
PTH, pg/mL >68 (%)	62 [46.1–81.2] 33 (37.5)	50.4 [35.6–70.4] 20 (26.3)	43.9 [35.4–55.3] 4 (9.5)	<0.01 <0.01

PTH, parathyroid hormone. Values are reported as numbers and percentages or as means with standard deviation.

**Table 4:** Logistic regression model for the associations of vitamin D deficiency in AUD patients.

Variable	Univariate (OR and 95%CI)	*p*-Value	* Multivariable (OR and 95%CI)	*p*
Age > 50	2.16 (1.12–4.18)	0.02	1.55 (0.77–3.11)	0.21
Impaired fasting glucose	2.66 (1.17–6.01)	0.02	2.51 (1.02–6.17)	0.04
FIB-4 > 3.25	4.55 (1.34–15.34)	0.01	4.27 (1.21–15.0)	0.02
GGT, U/L	1.00 (0.99–1.00)	0.09	-	
Creatinine, mg/dL	0.65 (0.38–1.10)	0.11	-	

* Adjusted by sex and alcohol consumption. Values are presented as odds ratios with 95% confidence intervals.

## Data Availability

The data presented in this study are available on request from the corresponding author due to privacy.

## Supplementary Materials

The following supporting information can be downloaded at: https://www.mdpi.com/article/10.3390/nu16081099/s1. STROBE Statement—Checklist of items that should be included in reports of cross-sectional studies.
